# Viewing photos and reading nouns of natural graspable objects similarly modulate motor responses

**DOI:** 10.3389/fnhum.2014.00968

**Published:** 2014-12-04

**Authors:** Barbara F. M. Marino, Miriam Sirianni, Riccardo Dalla Volta, Fabio Magliocco, Francesco Silipo, Aldo Quattrone, Giovanni Buccino

**Affiliations:** ^1^Dipartimento di Neuroscienze, Sezione di Fisiologia, Università di ParmaParma, Italy; ^2^Dipartimento di Psicologia, University Milano BicoccaMilano, Italy; ^3^IRCCS NeuromedPozzilli, Italy; ^4^Dipartimento di Scienze Mediche e Chirurgiche, Università “Magna Graecia” di CatanzaroGermaneto, Italy

**Keywords:** embodiment, language processing, canonical neurons, affordances, motor responses

## Abstract

It is well known that the observation of graspable objects recruits the same motor representations involved in their actual manipulation. Recent evidence suggests that the presentation of nouns referring to graspable objects may exert similar effects. So far, however, it is not clear to what extent the modulation of the motor system during object observation overlaps with that related to noun processing. To address this issue, 2 behavioral experiments were carried out using a go-no go paradigm. Healthy participants were presented with photos and nouns of graspable and non-graspable natural objects. Also scrambled images and pseudowords obtained from the original stimuli were used. At a go-signal onset (150 ms after stimulus presentation) participants had to press a key when the stimulus referred to a real object, using their right (Experiment 1) or left (Experiment 2) hand, and refrain from responding when a scrambled image or a pseudoword was presented. Slower responses were found for both photos and nouns of graspable objects as compared to non-graspable objects, independent of the responding hand. These findings suggest that processing seen graspable objects and written nouns referring to graspable objects similarly modulates the motor system.

## Introduction

It is known that hand-object interactions recruit a parieto-frontal circuit in the brain of both monkeys and humans subserving sensorimotor transformations (Rizzolatti et al., 1981, 1988, 2002; Kurata and Tanji, 1986; Taira et al., 1990; Hepp-Reymond et al., 1994; Jeannerod et al., 1995; Sakata et al., 1995; Binkofski et al., 1999; Grol et al., 2007; Hecht et al., 2013). Also the mere observation of objects that have the potential for being manipulated has been proven to be effective in modulating the activity of the motor system. Single-unit recording studies in monkeys have shown that a set of neurons known as “canonical neurons” discharges during the presentation of graspable objects (Rizzolatti et al., 1988; Murata et al., 1997; Raos et al., 2006; Umiltà et al., 2007). In keeping with this, brain imaging studies have shown the activation of fronto-parietal areas in the human brain during the observation of graspable objects (Chao and Martin, 2000; Grèzes et al., 2003a,b). The recruitment of the motor system during object observation is fine-tuned with the intrinsic features of objects that make them appropriate for manual action: for example motor evoked potentials (MEPs) recorded during the observation of graspable objects (e.g., a mug) with a broken handle were significantly different from MEPs recorded during the observation of a complete object (Buccino et al., 2009).

As far as language is concerned, the embodiment approach claims that language processing involves the activation of the same sensorimotor neural substrates recruited when one experiences the content of language material (Lakoff, 1987; Glenberg, 1997; Barsalou, 1999; Pulvermueller, 2001; Gallese, 2003; Gallese and Lakoff, 2005; Zwaan and Taylor, 2006; Fischer and Zwaan, 2008; Jirak et al., 2010). In recent years, there has been growing experimental evidence in favor of the embodiment. Much of this evidence comes from studies that used action verbs (individually presented or embedded in sentences) as stimuli (e.g., Pulvermueller et al., 2001, 2005; Hauk et al., 2004; Buccino et al., 2005; Tettamanti et al., 2005). Some works investigating the recruitment of the motor cortex during noun processing showed a modulation of the motor system activity according to manipulability of objects expressed by nouns (Glover et al., 2004; Tucker and Ellis, 2004; Lindemann et al., 2006; Myung et al., 2006; Bub et al., 2008; Cattaneo et al., 2010; Gough et al., 2012). Recently, slower hand motor responses have been shown during processing of nouns referring to hand-related objects (Marino et al., 2013; see also Sato et al., 2008; Dalla Volta et al., 2009 for similar results with verbs). Summing up, the studies reviewed so far clearly show that manipulation and observation of objects as well as processing of nouns referring to graspable objects modulate the activity of the motor system. It is not clear to what extent the modulation of the motor system during object observation overlaps with that related to noun processing. For example there is evidence that when some features of objects like the spatial location or the orientation are taken into account, then processing photos depicting graspable objects or nouns referring to those same objects differently modulate motor responses (Ferri et al., 2011; Myachykov et al., 2013).

Using a go-no go paradigm, we compared motor responses given while observing photos of graspable and non-graspable natural objects with those given while reading nouns of objects from the same categories. Given some evidence showing that tools and natural objects are differently represented in the brain and differently modulate the activity of the motor system (Boronat et al., 2005; Peeters et al., 2009; Rueschemeyer et al., 2010; Gough et al., 2012; Orban and Rizzolatti, 2012), we restricted our choice to natural objects. The experimental hypothesis was that if object and noun processing share the same neural substrates, as maintained by the embodiment approach, then objects and nouns should also exert a similar modulation of motor responses. In details, based on previous studies where a similar paradigm was used (e.g., Buccino et al., 2005; Sato et al., 2008; Marino et al., 2013), we expected slower motor responses for both types of stimuli with an early go-signal (150 ms). In Experiment 1 participants responded with the right hand while in experiment 2 participants responded with the left hand.

## Methods

### Participants

Forty (23 females; mean age = 22 years and 9 mo) and 43 (21 females; mean age = 23 years and 6 mo) undergraduate students from the University of Catanzaro took part in Experiment 1 and Experiment 2, respectively. They were right-handed according to the Edinburgh Inventory (Oldfield, 1971). None took part in both experiments. All participants were native Italian speakers, had normal or corrected-to-normal vision, and reported no history of language disorders. They were unaware of the purpose of the experiments and gave their informed consent before testing. The study was approved by the local Ethics Committee and conducted in accordance with the World Medical Organization (1996) and the procedure recommended by the Italian Association of Psychology (AIP).

### Stimuli

**Figure 1:**
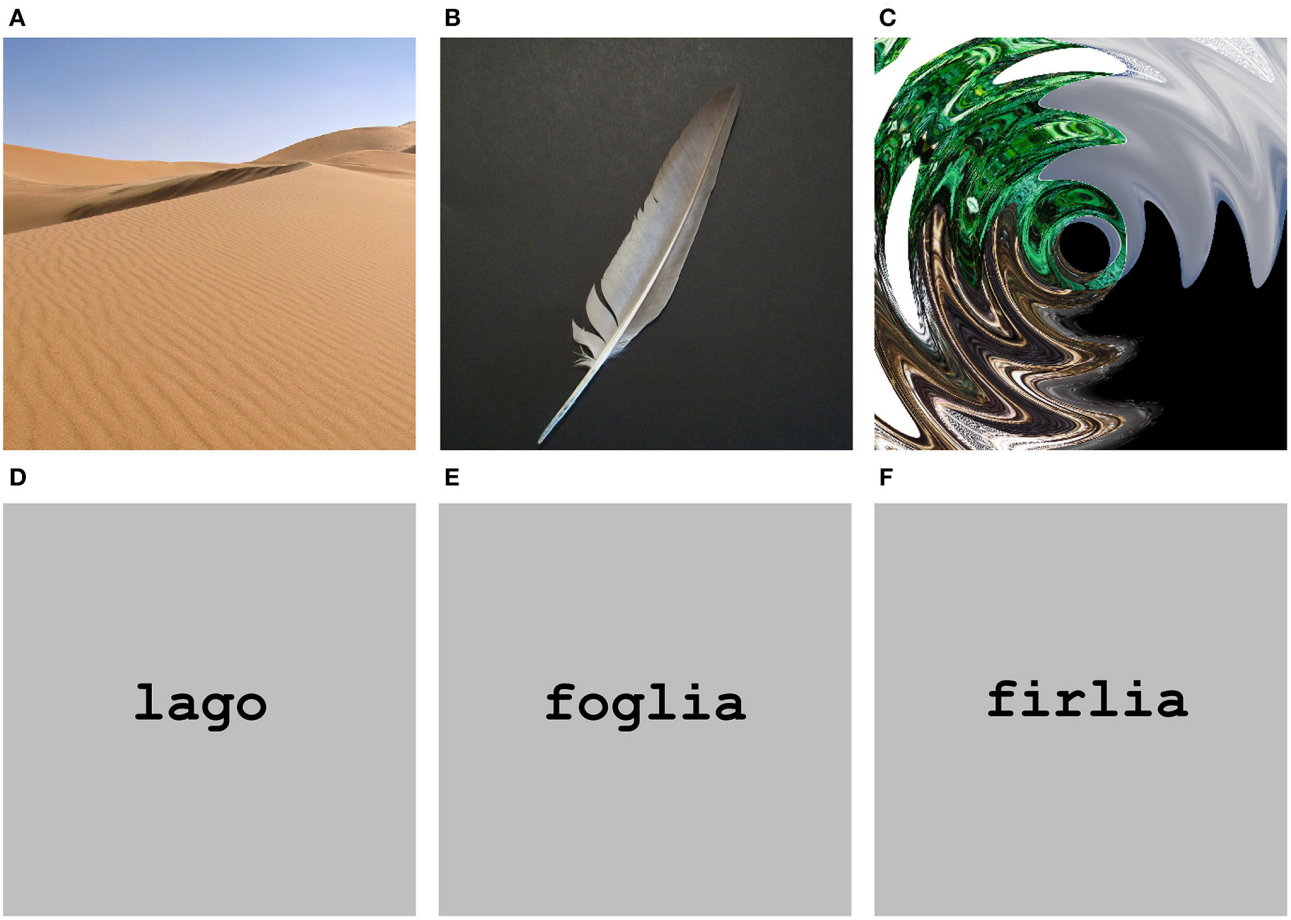
**Stimuli**. Examples of stimuli presented in the two experiments. Upper row shows visual items while lower row shows verbal items. **(A)** Non-graspable object. **(B)** Graspable object. **(C)** Scrambled image. **(D)** A noun expressing a non-graspable object. **(E)** A noun expressing a graspable object. **(F)** Pseudoword.

Forty Italian nouns (see Table A1) referring to natural objects and 40 pseudowords as well as 40 digital color photos (see Table A2) depicting natural objects and 40 scrambled images were used as stimuli. Twenty nouns referred to natural graspable objects (e.g., “foglia,” “leaf”) and 20 to natural non-graspable objects (e.g., “nuvola,” “cloud”). Figure 1 shows an example of each category. Nouns in the 2 categories were matched for word length [average values for nouns referring to graspable and non-graspable objects: 6.35 and 5.95; *F*_(1, 38)_ = 0.68, *p* = 0.41], syllable number [average values: 2.5 and 2.6, *F*_(1, 38)_ = 0.24, *p* = 0.63] and written lexical frequency [average values: 3.92 and 5.05 number of occurrences per million in Google search engine *F*_(1, 38)_ = 0.31, *p* = 0.58; average values: 6.13 and 6.95 number of occurrences per million in CoLFIS (Corpus e Lessico di Frequenza dell'Italiano Scritto ~3.798.000 words)—Laudanna et al., 1995—*F*_(1, 38)_ = 0.08, *p* = 0.78; r_Google/CoLFIS_ = 0.83, *p* < 0.0001]. Pseudowords were built by substituting one consonant and one vowel in two distinct syllables of each noun (e.g., “n*ip*ola” instead of “n*uv*ola”). With this procedure, pseudowords contained orthographically and phonologically legal syllables for the Italian language. In addition, nouns and pseudowords were matched for word length.

Photos depicted 20 graspable objects and 20 non-graspable objects. Figure 1 shows an example of each category. The scrambled images were built by applying Adobe Illustrator distorting graphic filters (e.g., *twist* and *zigzag*) to the photos depicting both graspable and non-graspable objects so to make them unrecognizable and then meaningless. All photos and scrambled images were 440 × 440 pixels. The nouns of objects depicted in the photos and the 40 Italian nouns used as stimuli were matched for word length [average values for visual items and for verbal item: 6.45 and 6.15; *F*_(1, 78)_ = 0.82, *p* = 0.37], syllable number [average values: 2.57 and 2.55; *F*_(1, 78)_ = 0.04, *p* = 0.84] and written lexical frequency [Google average values: 4.98 and 4.49; *F*_(1, 78)_ = 0.10, *p* = 0.75; CoLFIS average values: 7.74 and 6.54; *F*_(1, 78)_ = 0.18, *p* = 0.67]. For further analysis on the stimuli, see also Supplementary Materials. The same set of stimuli served both Experiment 1 and 2.

### Experimental design and procedure

The experiment was carried out in a sound-attenuated room, dimly illuminated by a halogen lamp directed toward the ceiling. Participants sat comfortably in front of a PC screen (LG 22″ LCD, 1920 × 1080 pixel resolution and 60 Hz refresh rate). The eye-to-screen distance was 60 cm.

**Figure 2:**
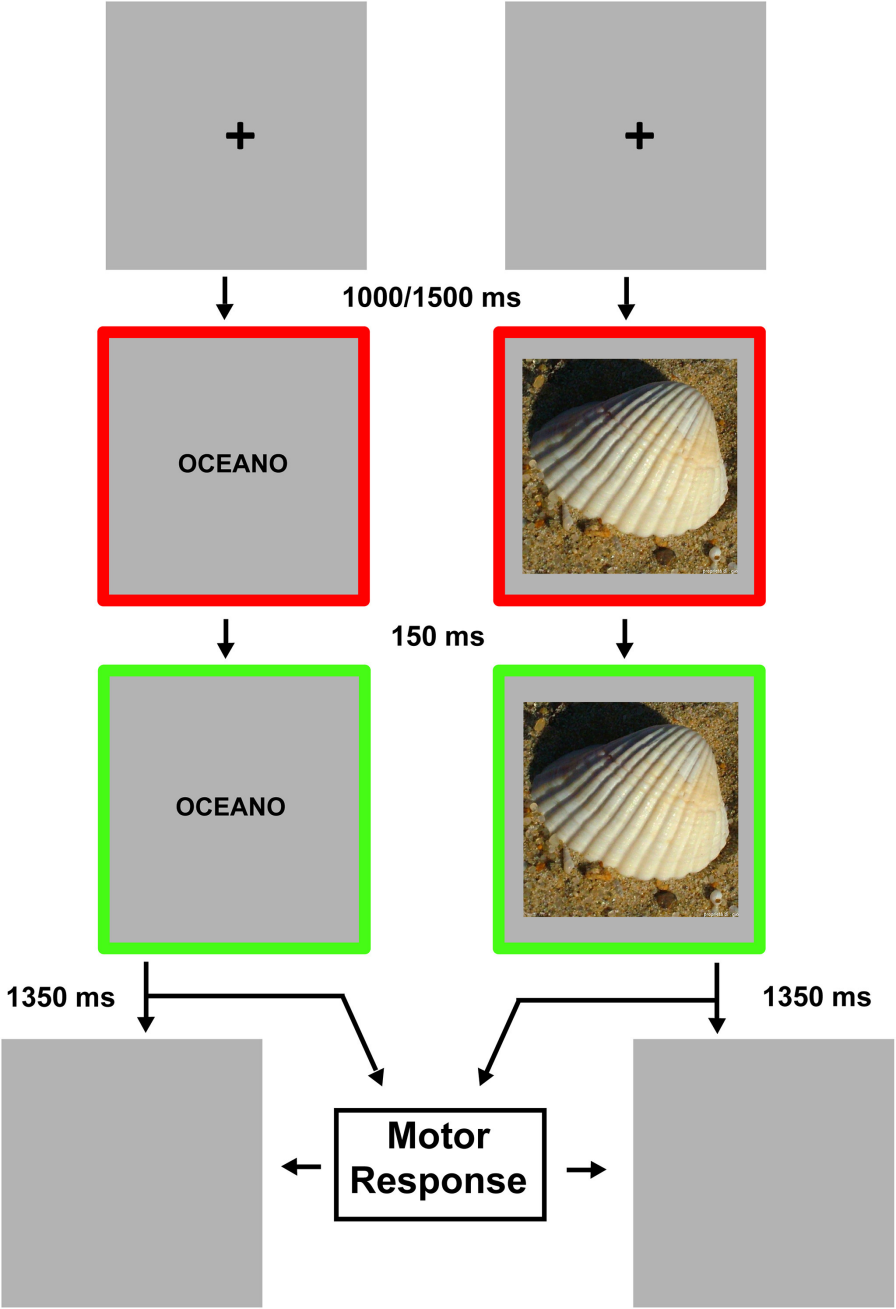
**Experimental procedure**. The timeline relative to the verbal stimuli presentation is depicted in the left part of the figure while the timeline relative to the visual stimuli presentation is depicted in the right part. Each trial started with a fixation cross. The appearance of the green frame represented the go-signal. Stimuli remained visible until motor response was given or 1500 ms had elapsed.

Figure 2 shows the experimental procedure. Each trial started with a black (RGB coordinates = 0, 0, 0) fixation cross displayed at the center of a gray (RGB coordinates = 178, 178, 178) background. After a delay of 1000–1500 ms (in order to avoid response habituation), the fixation cross was replaced by a stimulus item, either a noun/pseudoword or a photo/scramble. Note that the delay could be at any time between 1000 and 1500 ms. Trial-by-trial a value between 1000 and 1500 was picked according to a uniform distribution. The verbal labels were written in black lowercase Courier New bold (font size = 24). Stimuli were centrally displayed and surrounded by a red (RGB coordinates = 255, 0, 0) 20 pixels-wide frame. The red frame changed to green (RGB coordinates = 0, 255, 0) 150 ms after the stimulus onset. The color change of the frame was the “go” signal for the response. Participants were instructed to give a motor response, as fast and accurate as possible, by pressing a key on a computer keyboard centered on participants' body midline with their right (Experiment 1) or left (Experiment 2) index finger. They had to respond when the stimulus referred to a real object, and refrain from responding when it was meaningless (go-no go paradigm). After the go signal, stimuli remained visible for 1350 ms or until participant's response. Stimulus presentation and response times (RTs) collection were controlled using the software package E-Prime 2 (Psychology Software Tools, Inc.).

The experiment consisted of 1 practice block and 1 experimental block. In the practice block, participants were presented with 16 stimuli (4 photos of graspable/non-graspable objects, 4 scrambled images, 4 nouns of graspable/non-graspable objects and 4 pseudowords) which were not used in the experimental block. During the practice block, participants received feedback (“ERROR”) after giving a wrong response (i.e., responding to a meaningless or refraining from responding to a real item), as well as for responses given prior to go signal presentation (“ANTICIPATION”), or later than 1.5 s (“YOU HAVE NOT ANSWERED”). In the experimental block, the 160 items selected as stimuli were randomly presented with the constraint that no more than three items of the same kind (verbal, visual) or referring to objects of the same category (graspable, non-graspable, meaningless) could be presented on consecutive trials. No feedback was given to participants. Thus, the experiment, which lasted about 20 min, consisted of 80 go trials (40 nouns of objects, 50% graspable and 50% non-graspable, plus 40 photographs of objects, 50% graspable and 50% non-graspable) and 80 no-go trials (40 pseudowords plus 40 scrambled images), and 16 practice trials, for a total of 176 trials. To sum up, the experiment used a 2 × 2 repeated measures factorial design with Object Graspability (graspable, non-graspable) and Stimulus Type (nouns, photos) as within-subjects variables.

## Results

**Figure 3:**
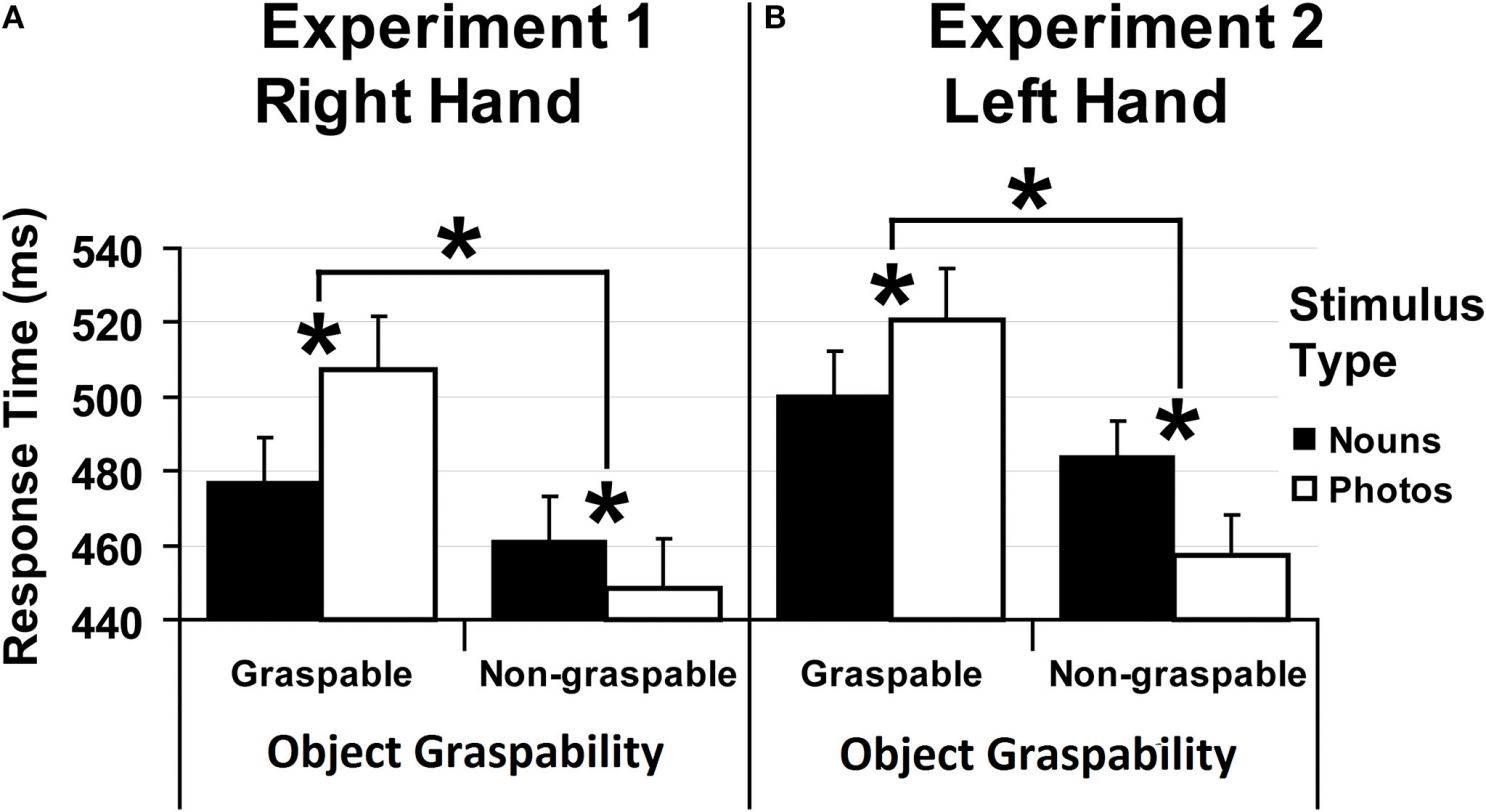
**Results**. Median values of response times collected in Experiment 1 **(A)** and in Experiment 2 **(B)** as a function of Object Graspability (graspable objects vs. non-graspable objects), separately for each Stimulus Type (nouns: black columns vs. photos: white columns). Error bars represent the confidence interval at 95%. Significant differences between values are marked by asterisks.

Figure 3 shows the result for both experiments.

Experiment 1. Trials with errors were excluded without replacement. Errors were not further analyzed given they were extremely rare (<5%). One participant was excluded from the analysis because his error rate exceeded 10%. RTs below 130 ms or above 1000 ms were omitted from the analysis (outliers). This cut-off was established so that no more than 0.5% of correct RTs were removed (Ulrich and Miller, 1994).

Median values of remaining RTs were calculated for each combination of Object Graspability (graspable and non-graspable) and Stimulus Type (photo and noun). These data entered a 2-way repeated measures analysis of variance (ANOVA) with Object Graspability and Stimulus Type as the within-subjects factors. *Post-hoc* comparisons were performed using the Newman-Keuls test with an alpha level of 0.05. Partial eta square values (η^2^_*p*_) are reported as an additional metric of effect size for all significant ANOVA contrasts.

The ANOVA revealed a main effect Object Graspability [*F*_(1, 38)_ = 73.90, *p* < 0.0001, η^2^_*p*_ = 0.66], reflecting slower responses for stimuli related to graspable objects (492 ms) as compared to those related to non-graspable objects (455 ms). Also the interaction between Object Graspability and Stimulus Type [*F*_(1, 38)_ = 25.01, *p* < 0.0001, η^2^_*p*_ = 0.40] was significant (Figure 3A). *Post-hoc* analysis showed that responses given to nouns referring to graspable objects were slower than responses to nouns referring to non-graspable objects (477 vs. 461 ms, *p* < 0.02). Similarly, responses given to photos referring to graspable objects were slower than those given to photos referring to non-graspable objects (507 vs. 448 ms, *p* < 0.0002). Moreover, responses to graspable objects were faster with nouns than with photos (477 vs. 507 ms, *p* < 0.0002) and, vice versa, for responses to non-graspable objects (nouns = 461 ms vs. photos = 448 ms, *p* < 0.04).

Experiment 2. Trials with errors and with outlier RTs were removed from the analysis as in Experiment 1. Four participants were excluded because their error rate exceeded 10%. Median values of correct RTs were computed and analyzed as in Experiment 1. The analysis revealed a main effect Object Graspability [*F*_(1, 38)_ = 48.50, *p* < 0.0001, η^2^_*p*_ = 0.56], reflecting slower responses for stimuli related to graspable objects (510 ms) as compared to those related to non-graspable objects (470 ms). Also the interaction between Object Graspability and Stimulus Type [*F*_(1, 38)_ = 21.94, *p* < 0.0001, η^2^_*p*_ = 0.37] was significant (Figure 3B). *Post-hoc* analysis showed that responses given to nouns referring to graspable objects were slower than responses to nouns referring to non-graspable objects (500 vs. 484 ms, *p* < 0.03). Similarly, responses given to photos referring to graspable objects were slower than those given to photos referring to non-graspable objects (521 vs. 457 ms, *p* < 0.0002). Moreover, responses to graspable objects were faster with nouns than with photos (500 vs. 521 ms, *p* < 0.007) and, vice versa, for responses to non-graspable objects (nouns = 484 ms vs. photos = 457 ms, *p* < 0.001).

## Discussion

In the present study, participants gave slower motor responses when they were presented with natural graspable objects as compared to natural non-graspable objects. This was true for both nouns and photos. As for nouns, these findings are in keeping with previous data concerning verbs (Buccino et al., 2005; Boulenger et al., 2006; Sato et al., 2008; Dalla Volta et al., 2009; De Vega et al., 2013, 2014), hand-related relative to foot-related nouns (Marino et al., 2013) and adjectives (Gough et al., 2013). To solve the requested semantic task in the case of nouns referring to graspable objects, it is most likely that participants relied on the motor representations of potential hand interactions with the object expressed by the verbal label. In this way, the motor system was engaged in two tasks at the same time, that is processing language material and performing a motor response (pressing the button). Hence participants paid a cost as revealed by a slowing down of their responses. It is worth underlining that our findings are not at odds with EEG and MEG studies (for review see Pulvermueller et al., 2009) supporting an early recruitment of the motor system during language processing and possibly a specific role of this system in this function. Thus, they seem to bolster this argument by showing that when the motor system is crucially involved in both a linguistic and a motor task there is a competition for resources. Moreover, our results concerning nouns are not in contrast with studies showing faster motor responses during processing of language material compatible with the direction of movement (Glenberg and Kaschak, 2002; Kaschak and Borreggine, 2008) or the type of prehension (e.g., Tucker and Ellis, 2004) required to give responses (i.e., the so-called Action Compatibility Effect, ACE). Indeed, this facilitation has been interpreted as an outcome that emerges relatively late in the time course of language processing (Taylor and Zwaan, 2008). In fact, the modulation of the motor system during language processing may change over time, moving from an early interference (operating between 100–200 ms after stimulus onset) to a later facilitation (operating later than 200 ms from stimulus presentation). The former effect could be a consequence of the fact that the motor system is a common neural substrate for action performance and language processing, while the latter may reflect priming triggered by the content of language material (for a computational model, see Chersi et al., 2010).

As for photos, it is well-accepted that the visual presentation of a graspable object automatically recruits motor representations of potential actions that the object affords to the observer (Gibson, 1977). We suggest that the recruitment of the motor system during the presentation of photos was relevant and most likely crucial to perform our semantic task, at least for graspable objects. As in the case of nouns, since the motor system was involved both in solving the semantic task and in planning and implementing the motor response, participants were slower when processing graspable objects. Similar findings were reported in a recent paper (Salmon et al., 2014). The authors found slower responses for photos depicting graspable as compared to non-graspable objects during a categorization task. In the present study this interference effect was stronger for photos than for nouns. This difference may be due to the fact that through photos the intrinsic features of objects, relevant for action, are immediately evident and specific (i.e., pertinent to the particular seen object) while through nouns these features are not related to specific objects but rather to a prototype of the class the objects belong to, most likely presented in a decontextualized fashion. It is worth stressing that even within language material it has been shown that the degree of sensorimotor specificity expressed by sentences affects how deeply the motor system is recruited during language processing (Marino et al., 2012).

At odds with a previous paper concerning nouns (Marino et al., 2013) where an interference effect was found only for responses given with the right hand, the present study did not find any difference between responses given by the two hands. In the study of Marino and colleagues, the authors suggested that the differential pattern of interference may be explained by the fact that only the left hemisphere is involved in both the linguistic and motor tasks, with the right one involved in only the motor task. Unfortunately, this explanation cannot account for the present results. We therefore forward that the different results in the two studies may be due to the kind of stimuli used. In fact, while Marino and colleagues used only nouns referring to tools, here we used nouns referring to natural objects. It is well known that tools and natural objects are differently represented in the brain (Boronat et al., 2005; Peeters et al., 2009; Rueschemeyer et al., 2010; Gough et al., 2012; Orban and Rizzolatti, 2012) and in particular, a specific sector of the left inferior parietal lobule is devoted to tool use in humans. It may be argued therefore, that besides the linguistic role of the left hemisphere the different modulation of the two hemispheres in the paper of Marino et al. (2013) is due to the specific role of the left hemisphere in processing tools and in praxic functions (Heilman et al., 1982; De Renzi and Lucchelli, 1988; Buxbaum and Kalénine, 2010).

Taken as a whole, our data support that semantic processing of visually presented graspable objects and nouns referring to the same object category is sub-served by common neural substrates crucially involving the motor system (Ganis et al., 1996; Vandenberghe et al., 1996; Van Doren et al., 2010). A similar modulation of the motor system has been also assessed for visually presented actions and verbs (Aziz-Zadeh et al., 2006; Baumgaertner et al., 2007; De Vega et al., 2014). Recently, Borghi and Riggio (2009) proposed a distinction between stable and temporary affordances of objects, the former being related to features like shape and size, the latter being related to aspects like orientation and position. One plausible explanation for the present findings is that a similar modulation of the motor system during processing of both nouns and photos occurred because, given the task, only stable affordances of objects were coded. In keeping with this explanation, there is evidence that when temporary affordances, such as the position or the orientation, come into account then photos and nouns differently modulate the activity of the motor system (Ferri et al., 2011; Myachykov et al., 2013). An alternative but not mutually exclusive explanation may be related to the kind of stimuli used. As compared to previous studies that in most cases employed tools (or a combination of both tools and natural objects) in the present study we used only natural objects. For this kind of objects it is less clear cut which part of the object can elicit hand actions and it is hard to disentangle between manipulation and function knowledge of objects (Boronat et al., 2005). Indeed information about the position or the orientation of an object may be more relevant when using a hammer rather than when grasping an apple.

### Conflict of interest statement

The authors declare that the research was conducted in the absence of any commercial or financial relationships that could be construed as a potential conflict of interest.

## Appendix

**Table A1:** **List of the Italian nouns used in Experiment 1 and 2, their English translation, graspability of their referents, lexical frequency (number of occurrence per million in Google search engine—e.g., Marino et al., 2012—and in CoLFIS search engine—Laudanna et al., 1995), length and syllable number**.

**Italian Noun**	**English Translation**	**Referent Graspability**	**Lexical frequency Google/COLFIS**	**Word Length/Syllable**
Bulbo	Bulb	Yes	2.17/0.13	5/2
Pigna	Pinecone	Yes	1.14/0.08	5/2
Bocciolo	Bud	Yes	0.42/0.39	8/3
Corteccia	Bark	Yes	0.58/2.82	9/3
Foglia	Leaf	Yes	3.32/6.79	6/2
Fossile	Fossil	Yes	1.63/0.01	7/3
Cuoio	Leather	Yes	3.52/13.0	5/3
Granello	Grain	Yes	0.33/0.26	8/3
Neve	Snow	Yes	34.4/35.8	4/2
Paglia	Straw	Yes	2.42/6.11	6/2
Pepita	Nugget (gold)	Yes	1.99/0.00	6/3
Picciolo	Stalk	Yes	0.61/0.22	8/3
Pietra	Stone	Yes	11.7/28.6	6/2
Ramoscello	Sprig	Yes	0.40/0.19	10/4
Guscio	Shell (egg)	Yes	1.09/1.89	6/2
Sabbia	Sand	Yes	5.01/17.3	6/2
Scorza	Rind	Yes	1.11/0.80	6/2
Seme	Seed	Yes	4.92/6.39	4/2
Stelo	Stem	Yes	1.03/1.66	5/2
Sughero	Cork (bark)	Yes	0.69/0.29	7/3
Altopiano	Upland	No	0.87/1.25	9/4
Faglia	Fault (line)	No	0.09/0.15	6/2
Bosco	Wood (trees)	No	14.9/17.8	5/2
Caverna	Cavern	No	3.76/1.71	7/3
Collina	Hill	No	4.86/10.0	7/3
Cratere	Crater	No	0.54/0.33	7/3
Nuvola	Cloud	No	3.55/3.60	6/3
Frana	Landslide	No	3.66/0.15	5/2
Lago	Lake	No	3.55/16.3	4/2
Laguna	Lagoon	No	2.42/2.35	6/3
Masso	Boulder	No	1.38/0.10	5/2
Oasi	Oasis	No	3.32/4.55	4/2
Oceano	Ocean	No	10.8/10.4	6/3
Penisola	Peninsula	No	2.48/7.15	8/4
Foce	Mouth (river)	No	1.58/0.92	4/2
Cascata	Waterfall	No	3.51/1.85	7/3
Riva	Shore	No	17.3/13.8	4/2
Scoglio	Reef	No	0.98/2.00	6/2
Spiaggia	Beach	No	12.6/34.1	8/3
Valle	Valley	No	9.02/10.4	5/2

**Table A2:** **List of the Italian nouns (and their English translation) of the objects depicted in the photographs used in Experiment 1 and 2, their graspability, lexical frequency, length and syllable number**.

**Depicted object**		**Object Graspability**	**Lexical freq. Google/COLFIS**	**Word Length/syllable**
**Italian noun**	**English tr**.			
Buccia	Peel (fruit)	Yes	0.98/1.38	6/2
Carbone	Coal (lump)	Yes	13.5/8.69	7/3
Conchiglia	Shell	Yes	0.91/1.27	10/3
Corallo	Coral	Yes	2.12/0.76	7/3
Diamante	Diamond	Yes	2.70/1.71	8/3
Fiore	Flower	Yes	17.2/18.61	5/2
Creta	Clay	Yes	0.53/0.48	5/2
Ghianda	Acorn	Yes	0.48/0.04	7/2
Osso	Bone	Yes	5.46/5.41	4/2
Perla	Pearl	Yes	2.18/1.34	5/2
Petalo	Petal	Yes	0.53/0.02	6/3
Baccello	Husk	Yes	0.33/0.01	7/3
Piuma	Feather	Yes	1.55/1.38	5/2
Radice	Root	Yes	2.58/6.48	6/3
Sasso	Pebble	Yes	4.97/4.66	5/2
Spiga	Ear (wheat)	Yes	0.38/0.13	5/2
Ghiacciolo	Icicle	Yes	0.08/0.01	10/3
Legname	Timber	Yes	0.68/0.68	7/3
Muschio	Moss	Yes	0.57/0.91	7/3
Capelli	Hair	Yes	19.9/82.5	7/3
Albero	Tree	No	10.3/28.2	6/3
Ruscello	Brook	No	0.33/0.78	8/3
Canyon	Canyon	No	0.51/0.55	6/2
Radura	Glade	No	0.98/1.25	6/3
Cometa	Comet	No	1.29/0.11	6/3
Deserto	Desert	No	7.37/20.3	7/3
Scogliera	Cliff	No	0.87/1.46	9/3
Foresta	Forest	No	3.85/13.9	7/3
Ghiacciaio	Glacier	No	1.64/1.67	9/3
Grotta	Cave	No	2.80/6.80	6/2
Iceberg	Iceberg	No	1.25/1.03	7/2
Stella	Star	No	17.5/17.9	6/2
Lava	Lava	No	0.42/1.78	4/2
Luna	Moon	No	30.9/38.2	4/2
Vulcano	Volcano	No	4.84/3.08	7/3
Crinale	Ridge	No	0.98/0.93	7/3
Palude	Marsh	No	0.77/1.33	6/3
Pianeta	Planet	No	6.48/21.4	7/3
Pineta	Pine forest	No	2.71/0.20	6/3
Prato	Meadow	No	25.9/12.2	5/2
