# Supplementary material for: Viewing photos and reading nouns of natural graspable objects similarly modulate motor responses
Source: Front Hum Neurosci. 2014 Dec 4;8:968. doi: 10.3389/fnhum.2014.00968 (PMC4255516; doi:10.3389/fnhum.2014.00968)
Supplement: Supplementary file 1 [file DataSheet1.DOCX]

**Supplementary Material**

Preliminary validation of the digital photos

We named all objects depicted in the set of photos with the most appropriate verbal labels (nouns). The nouns we gave to objects in the photos and the 40 Italian nouns used as stimuli were matched for word length, syllable number and written lexical frequency. In order to assess whether the object depicted in a photo could be correctly identified and how well it represented that specific type of object, we asked 20 Italian students (12 female, mean age = 23 ys and 5 mo), different from those enrolled in the behavioral experiment, to observe each photo and give a name to the observed object. This procedure allowed us to assess to what extent the nouns we attributed overlapped with those provided by our students. Subsequently, they had to answer the following question: *How much the presented object is a good exemplar of that type of object?* (e.g., How much the presented shell represents the concept of shell)? This procedure allowed us to assess whether the presented object was a typical instance of that object. Ratings were given according to a Likert scale (1 = very little, 7 = a lot). The average score for each item entered a one-way repeated measures ANOVA with Object Graspability (2 levels, graspable vs. non-graspable) as the within-subject factor. Significance level was set at p = .05. Results showed that on average no objects were scored below 5, indicating that they were considered as good exemplars of the category they belonged to. In addition, there was no significant difference according to the Manipulability [average values for graspable and non-graspable objects: 6.17 and 6.12; F(1,19) = .25, p = .63].
